# Turnover Intention and Its Associated Factors Among Psychiatrists in 41 Tertiary Hospitals in China During the COVID-19 Pandemic

**DOI:** 10.3389/fpsyg.2022.899358

**Published:** 2022-06-10

**Authors:** Yating Yang, Ling Zhang, Mengdie Li, Xiaodong Wu, Lei Xia, Daphne Y. Liu, Tingfang Liu, Yuanli Liu, Feng Jiang, Yi-lang Tang, Huanzhong Liu, Nadine J. Kalow

**Affiliations:** ^1^Department of Psychiatry, Chaohu Hospital of Anhui Medical University, Hefei, China; ^2^Department of Psychiatry, School of Mental Health and Psychological Sciences, Anhui Medical University, Hefei, China; ^3^Department of Psychiatry and Behavioral Sciences, Emory University, Atlanta, GA, United States; ^4^Department of Psychological and Brain Sciences, Washington University in St. Louis, St. Louis, MO, United States; ^5^School of Health Policy and Management, Chinese Academy of Medical Sciences and Peking Union Medical College, Beijing, China; ^6^School of International and Public Affairs, Shanghai Jiao Tong University, Shanghai, China; ^7^Institute of Healthy Yangtze River Delta, Shanghai Jiao Tong University, Shanghai, China; ^8^Atlanta VA Medical Center, Decatur, GA, United States

**Keywords:** turnover intention, psychiatrists, COVID-19 pandemic, insomnia, burnout, job satisfaction

## Abstract

**Background:**

Turnover intention, an employee’s intention to voluntarily leave their jobs, affects workforce sustainability. However, scarce data are available about turnover intention and its associated factors among psychiatrists in China, especially during the COVID-19 pandemic. The current research was designed to address this gap.

**Methods:**

An anonymous, nationwide online survey was disseminated to psychiatrists in 41 psychiatric hospitals in China. We collected demographic data, job-related information (duration of employment, history of participation in the frontline work against COVID-19, job satisfaction), and participants’ turnover intention in the next 12 months.

**Results:**

In total, 3,973 psychiatrists completed the survey. The sample was predominantly female (58.8%) and married (79.5%), and nearly three-fourths (73.5%) had children. More than one-third (35.6%) had a master’s or doctoral degree in addition to their medical degree. The overall level of job satisfaction was moderate. The rate of turnover intention was 22.0% and was comparable in males and females (22.9% in males and 21.3% in females, respectively). Psychiatrists who had participated in the frontline work of COVID-19 were more likely to report an intention to leave their current job. Multiple regression analyses suggested that turnover intention was significantly associated with having insomnia, longer working hours, and more working days per week.

**Conclusion:**

During the pandemic period, approximately one-fifth of psychiatrists in China reported turnover intention. Factors associated with turnover intention included high job-related burdens, low job satisfaction, participation in the frontline work against COVID-19, and insomnia. To improve psychiatric workforce sustainability, policymakers and hospital administrators need to be aware of this potential challenge and address the concerns of psychiatrists in China.

## Introduction

Although much progress has been made in the past three decades, mental health resources in China are overall limited and unevenly distributed (Xia et al., 2021). A 2014 survey found that in China there were 1,650 mental health institutions, 228, 000 psychiatric beds (Liu et al., 2013), and more than 20,000 psychiatrists, the majority of whom are located in economically developed regions. The number of mental health specialists *per capita* in China is much lower than that in most developed countries, and even some developing countries. To make matters worse, many factors affect the sustainability of the current workforce, such as heavy workload, long working hours, low salaries, and low job satisfaction (Jiang et al., 2019a; Yao et al., 2021; Han et al., 2022).

Turnover intention, defined as an employee’s intention to voluntarily leave their jobs, is a good indicator of workforce sustainability. The high turnover intention rate of psychiatric professionals is a serious problem worldwide that has caused a global shortage of psychiatric professionals. For example, previous studies have found that psychiatrists in Australia (Alsaraireh et al., 2014; Scanlan and Still, 2019) and the United States have a high turnover intention. Similar data have been found in China, where 20% of psychiatrists endorsed an intention to terminate their employment before the COVID-19 pandemic (Jiang et al., 2018). Studies suggest that turnover intention predicts employees’ actual turnover behavior (Zhang and Feng, 2011). Therefore, it is of paramount importance to investigate the turnover intention among psychiatrists and to understand its associated factors. Existing studies related to this topic are limited. They were often conducted in single local hospitals (Huang et al., 2018; Peng et al., 2018; Hou, 2019), providing information only relevant to the turnover intention of psychiatrists in local areas. Other investigations were conducted before the COVID-19 pandemic, a global event that has profoundly impacted many aspects of healthcare.

The sudden outbreak of COVID-19 has placed tremendous demands on healthcare professionals, including increased workload, increased physical exertion, and lack of personal protective equipment, which can have a negative impact on their physical and mental health. Several studies found that the rates of depression and anxiety increased significantly during the pandemic period (Elbay et al., 2020; AL-Ghasab et al., 2021; Al-Humadi et al., 2021; Olashore et al., 2021; Rajabimajd et al., 2021; Tuna and Özdin, 2021). Beyond healthcare workers, the rates of depression and anxiety among ordinary citizens also increased markedly during the COVID-19 pandemic, which in turn increased the work pressure on psychiatrists (Guvenc et al., 2021; Liu C. et al., 2021; McLoughlin et al., 2021; Zhang et al., 2021a). The increasing job strain on psychiatrists may lead them to consider new career plans (turnover intention). Several studies have shown that the COVID-19 pandemic contributed to higher rates of turnover intention job among healthcare professionals in several countries (Cole et al., 2021; Hou et al., 2021; Labrague and de Los Santos, 2021). However, there is a lack of representative data on the prevalence and associated factors of psychiatrists’ turnover intention during the COVID-19 epidemic in China.

The survey in the present research was designed to investigate the turnover intention of psychiatrists in China, as well as its associated factors, during the COVID-19 pandemic. Our overall aim was to examine the turnover intention rate of psychiatrists during the COVID-19 pandemic. It was hypothesized that psychiatrists who participated in COVID-19 frontline work would be more likely to report the turnover intention than those who did not have such experience. Additionally, we hoped to identify some actionable areas that would inform policymakers on better resource allocation. Thus, we explored how other factors, such as job-related burdens and job satisfaction, are associated with turnover intention.

## Materials and Methods

### Design and Participants

The current study was part of the National Medical Service Improvement Project, sponsored by China’s National Health Commission. Its main purpose was to improve the quality of medical services and the working environment. The start date of this study was January 11, 2020 (the time when the first subject filled out the questionnaire), and the end date was March 15, 2020 (the time when the last subject filled out the questionnaire). All psychiatrists working in 41 tertiary psychiatric hospitals across 28 provinces were approached *via* WeChat to participate in a survey. To improve the response rate and ensure the accuracy of the information collected, the medical staff office of each participating hospital approached psychiatrists in those hospitals and encouraged them to complete the questionnaire. The staff of these hospitals did not have access to participants’ responses. WeChat is a popular social network mobile application in China, and each WeChat account can only complete one survey, which prevents repeated submissions. The survey was approved by the Ethics Committee of Hospital of Anhui Medical University. The approval number was 202002-kyxm-02.

### Data Collected

#### Demographic Data and Job-Related Factors

We collected participants’ demographic data including age, sex (female and male), marital status (single, married, divorced, or widowed), number of children, and highest education (college/medical, master’s, or doctorate). We also collected job-related factors including a history of participation in the frontline work during the COVID-19 pandemic (yes or no), the number of night shifts per month, working hours per day, working days per week, and duration of employment. In addition, the monthly income (RMBs) of medical staff was collected, which was categorized into three levels according to the Chinese standards: low: ≤5,000 RMBs/month; medium: 5,001–10,000 RMBs/month; and high: >10,000 RMBs/month (RMB: USD = 1: 6.36). We also categorized participants’ location of residency into economically developed regions and less developed regions, based on the level of economic development (Geng et al., 2020).

#### Turnover Intention, Job and Income Satisfaction, and Insomnia

Consistent with the extant literature (Fang et al., 2014; Van Bogaert et al., 2014; Jiang et al., 2019b), we assessed participants’ turnover intention in the past month using a single-item question (yes or no). The short version of the Minnesota Satisfaction Questionnaire (MSQ-SF) was used to assess job satisfaction (Weiss et al., 1967), which has 20 items with Likert-type scaling, ranging from 1 (very dissatisfied) to 5 (very satisfied). The Chinese version of the MSQ-SF has been widely used and has demonstrated good reliability and validity (Lu et al., 2016; Jiang et al., 2018; Zhou et al., 2019; Liu D. et al., 2021). The Cronbach’s alpha was 0.964 in this sample, suggesting high internal consistency. Participants were also asked whether they were satisfied with their income (yes or no). We assessed insomnia by asking participants to rate the frequency at which they experienced insomnia over the past 12 months using the following options: no insomnia, occasional insomnia (less than 2 times a month), insomnia sometimes (1–2 times a week), frequent insomnia (3–5 times a week), and insomnia almost every day. We grouped participants into two categories based on their responses: participants without insomnia (endorsed no insomnia) and those with insomnia (endorsed at least occasional insomnia).

### Statistical Analysis

The Kolmogorov–Smirnov one-sample test was used to determine whether each of the continuous variables was normally distributed. We conducted bivariate analyses to examine how turnover intention is associated with each variable of interest. For continuous variables with a normal distribution, we used Student’s *t*-tests to examine the difference between those who reported and did not report turnover intention, and for those variables with non-normal distribution, we used the Mann–Whitney U test to examine group differences. The chi-square test was used for categorical variables. Following bivariate analyses, a multiple logistic regression analysis was performed to examine the associations between turnover intention (outcome variable) and other variables (simultaneously entered as predictors in the model). This was done to examine which variables remain significant after accounting for other variables. Turnover intention being “no” was used as the reference level. We used SPSS23.0 for all data analyses. Statistical significance is reached at the *p* < 0.05 level.

## Results

### General Characteristics of Participants

A total of 4,899 psychiatrists in 41 tertiary hospitals were recruited to complete this survey and 3,973 did so (response rate = 81.10%). After removing 190 surveys with incomplete answers and logical errors, data from 3,783 psychiatrists throughout China were included in the final analyses. As shown in Table 1, participants were on average 38.78 years old (SD = 8.69), and the majority were married (79.5%) and had children (73.5%). Of all participants, 64.4% had a college/medical degree, 29.5% a master’s degree, and 6.1% a doctoral degree. The average length of employment was 14.08 years (SD = 9.71). More than one-fifth (22.4%) of the psychiatrists reported working on the front lines during the COVID-19 pandemic. The median monthly income level in this group was 12,144 RMBs (equivalent to 1908.65 $USD). For details, please see Table 1.

**Table 1:** Basic characteristics of psychiatrists in the final sample (*N* = 3,783).

Characteristic	*Mean*	*SD*
Age (years)	38.78	8.69
Duration of employment (years)	14.08	9.71
Working days per week (days)	5.58	0.62
Daily working hours (hours)	8.84	1.34
Number of night shifts per month	3.03	2.39
Job satisfaction	63.22	12.99
	*N*	%
Gender (male)	1,521	40.2
*Education level*		
Undergraduate/medical	2,438	64.4
Master’s degree	1,115	29.5
Doctoral degree	230	6.1
*Marital status*		
Single	631	16.7
Married	3,008	79.5
Divorced or widowed	144	3.8
With children (yes)	2,779	73.5
Regional distribution (developed regions)	2,063	54.5
Participated in the frontline work of COVID-19? (yes)	887	22.4
Insomnia (yes)	2,970	78.5
*Income*		
Low	485	12.8
Middle	1,558	41.2
High	1,740	46.0
Turnover intention (yes)	831/3,783	21.9

### Turnover Intention, Job Satisfaction, and Insomnia

Overall, 22.0% of all participants reported an intention to leave their current job. The overall job satisfaction was moderate (63.22 ± 12.99 on the MSQ). Nearly 4/5 of psychiatrists (78.5%) reported insomnia with varying frequencies, and only about 1/5 reported no insomnia.

### Bivariate Analysis of Psychiatrist’s Turnover Intention

As shown in Table 2, psychiatrists who had an intention to leave reported significantly more working days per week, working hours per day, and night shifts compared to those without an intention to leave (all value of *p* < 0.05). A turnover intention was also more likely to be reported in those who lived in less developed areas, had lower income, reported lower job satisfaction (MSQ-SF score), and were less satisfied with their income (all value of *p* < 0.05). Importantly, the experience of participating in frontline work during the COVID-19 pandemic was also a significant predictor of turnover intention (*χ*^2^ = 11.057, *p* = 0.001).

**Table 2:** Comparison of socio-demographic characteristics and work-related factors between of psychiatrists with intention and without intention to leave.

Characteristic	With intention to leave (*N* = 831)	Without intention to leave (*N* = 2,952)	*X^2^/Z*	*p*
Age (years)	38.20 ± 8.03	38.95 ± 8.87	−1.37	0.17
*Gender (%)*				
Male	349 (22.9)	1,172 (77.1)	1.422	0.233
Female	482 (21.3)	1,780 (78.7)		
*Education level (%)*				
College/medical degree	2,409 (20.9)	9,128 (79.1)	7.55	**0.023**
Master’s degree	242 (20.1)	964 (79.9)		
Doctorate degree	40 (17.2)	193 (82.8)		
*Marital status (%)*				
Single	162 (25.7)	469 (74.3)	33.517	**<0.001**
Married	228 (20.4)	887 (79.6)		
Divorced or widowed	38 (16.5)	192 (83.5)		
*With children*				
No	228 (22.7)	776 (77.3)	0.440	0.507
Yes	603 (21.7)	2,176 (80.5)		
*Regional distribution (%)*				
Developed regions^a^	382 (18.5)	1,681 (81.5)	32.506	**<0.001**
Less developed regions^b^	449 (26.1)	1,271 (73.9)		
*Participated in the frontline work of COVID-19? (%)*				
Yes	230 (25.9)	657 (74.1)	11.057	**0.001**
No	570 (20.6)	2,194 (79.4)		
Duration of employment (years)	13.44 ± 8.84	14.25 ± 9.94	−1.18	0.236
Working days per week	5.73 ± 0.64	5.54 ± 0.61	−8.37	**<0.001**
Daily working hours	9.17 ± 1.39	8.74 ± 1.31	−8.50	**<0.001**
Number of night shifts per month	3.57 ± 2.50	2.88 ± 2.34	−7.32	**<0.001**
Job satisfaction (MSQ-SF)	53.19 ± 11.85	66.07 ± 11.84	−25.22	**<0.001**
*Insomnia (%)*				
No	217 (8.8)	282 (74.5)	98.08	**<0.001**
Yes	75 (9.2)	738 (90.8)		
*Income^c^*				
Low	136 (28.0)	349 (72.0)	51.55	**<0.001**
Middle	403 (25.9)	1,155 (74.1)		
High	292 (16.8)	1,448 (83.2)		
*Satisfied with income (%)*				
Yes	92 (6.7)	1,275 (93.3)	289.89	**<0.001**
No	739 (30.6)	1,677 (69.4)		

a Developed regions: Guangdong, Jiangsu, Shandong, Zhejiang, Henan, Sichuan, Hubei, Hunan, Hebei, Fujian, Shanghai, Beijing, and Anhui.

b Less developed regions: Liaoning, Shaanxi, Jiangxi, Chongqing, Guangxi, Tianjin, Yunnan, Inner Mongolia, Shanxi, Heilongjiang, Jilin, Guizhou, Xinjiang, Hainan, Ningxia, and Qinghai.

c Monthly income, low: ≤5,000, medium: 5,000–10,000, or high: >10,000 RMBs, one dollar is approximately equal to 6.95 RMBs at the time of study.

Bolded values of *p* < 0.05.

### Factors Associated With Turnover Intention

Multiple logistic regression analysis including all predictors suggested that turnover intention was significantly associated with higher workload (i.e., working days per week and working hours per day), and lower job satisfaction. Furthermore, we found that those with insomnia were 2.26 times more likely to report turnover intention than those without insomnia (OR = 2.261, 95% CI = 1.693–3.020). Additionally, those who participated in the frontline work during the COVID-19 pandemic had a higher turnover intention rate than those who did not do so (OR = 1.239, 95% CI = 1.011–1.519 see Table 3).

**Table 3:** Logistic regression analysis of factors associated with turnover intention in psychiatrists.

	*p*	OR	95% CI	
			Lower	Upper
Age (years)	0.613	0.988	0.943	1.035
Working years (years)	0.967	1.001	0.960	1.044
Gender (reference: female)	0.518	1.064	0.882	1.283
Regional distribution (reference: developed regions)	0.165	1.150	0.944	1.401
With children	0.287	1.196	0.860	1.663
Working days per week	**0.012**	1.223	1.045	1.432
Daily working hours	**0.002**	1.118	1.042	1.199
Number of night shifts per month	0.592	1.011	0.970	1.054
*Income (reference: high)*				
Middle	0.101	1.198	0.966	1.486
Low	0.079	1.315	0.968	1.786
*Education level (reference: college/medical degree)*				
Add on master’s degree	0.989	1.002	0.799	1.256
Add on doctorate degree	0.471	0.841	0.525	1.347
*Marital status (reference: married)*				
Single	**0.019**	1.550	1.075	2.237
Divorced or widowed	0.170	1.370	0.874	2.147
*Participated in the frontline work of COVID-19? (reference: no)*				
Yes	**0.039**	1.239	1.011	1.519
Job satisfaction (MSQ-SF)	**<0.001**	0.916	0.908	0.924
*Insomnia (reference: no insomnia)*				
Insomnia	**<0.001**	2.261	1.693	3.020
Constant	0.033	5.943		

Bolded values of *p* < 0.05. OR, odds ratio.

## Discussion

The current study is the first to investigate the turnover intention of Chinese psychiatrists during the period of the COVID-19 pandemic. The sample was large and nationally representative, as it included psychiatrists from 41 tertiary hospitals in China. Our findings were concerning as approximately one-fifth (21.97%) of psychiatrists in China planned to leave their current jobs within 1 year. Factors associated with turnover intention included high job-related burdens, such as work hour demands, participation in frontline work, and insomnia.

### Investigation of the Turnover Intention of Psychiatrists

The high rate of turnover intention in our sample is similar to findings from previous studies (Jiang et al., 2018; Zhou et al., 2018). For example, a 2017 study by Tang et al. found that 20.2% of psychiatric nurses plan to leave within 12 months (Jiang et al., 2019b). Surprisingly, the turnover intention rate found in our study was much lower than that of general practitioners who had a turnover intention rate of 78% according to a 2018 study (Gan et al., 2018). The large difference between the findings of these two studies may be attributed to how turnover intention was measured. Specifically, the 2018 study used the turnover intention scale that consisted of six items assessing participants’ desire to change their job within the last 12 months, which investigated not only whether participants were willing to leave their current job, but also whether they would change careers after leaving (the latter was of less interest to the current investigation). Instead, we used a single-item binary question to directly assess whether there was a turnover intention in the past month. Previous studies have shown that the use of a single binary measure of turnover intention can be a good indicator of turnover intention in a group (Fang et al., 2014; Jiang et al., 2019b). Additionally, we judge that asking participants to reflect on their actual turnover intention over the past month involves less biased reporting compared to asking participants to anticipate their behaviors in the future (e.g., participants might overestimate their willingness to leave their job when asked to predict their future intentions). On the other hand, our lower rate of turnover intention may be explained by our sample of psychiatrists in tertiary hospitals in China, where the income and promotion opportunities are much more desirable than those of primary health workers.

### Association of Turnover Intention With Participating in Frontline COVID-19 Patient Care

Further, psychiatrists who took part in the frontline work against COVID-19 were more likely to have the idea of leaving their employment than those who did not do so. The COVID-19 pandemic is a historic event that has a broad and profound impact on every citizen. Following the outbreak of COVID-19, many Chinese doctors joined the frontline work in the fight against COVID-19. Doctors who took part in the frontline work against COVID-19 had a heavier workload and burden and showed higher rates of anxiety (Uyaroğlu et al., 2020; Mattila et al., 2021; Bohlken et al., 2022) and depression (Yigitoglu et al., 2021; Zhang et al., 2021b) compared to their counterparts who did not assume such responsibilities. Thus, participating in the COVID-19 frontline work is an important factor leading to an increase in doctors’ turnover intention. For example, the results of this study showed that 25.9% of COVID-19 frontline doctors reported having the idea of leaving their employment, which was significantly higher than that of the doctors who were not involved in the fight against COVID-19 (20.6%). In addition, another survey showed that during the COVID-19 pandemic, about 1/4 of American medical workers considered leaving their current jobs in the near future (USA Today/Ipsos survey of Health Care Worker, 2022). A 2017 survey conducted by Jiang et al. (2018) showed that 20% of Chinese psychiatrists had the idea of leaving (Zhou et al., 2018). However, the turnover intention of the psychiatrists involved in the frontline work against COVID-19 appears to be slightly higher than that of the previous survey. This may be due to the relatively high unemployment rate during the pandemic, and thus available alternative job options are limited. As such, doctors may not be able to find their next job quickly after leaving their job.

### Association of Turnover Intention With Job Satisfaction

The MSQ-SF score (job satisfaction) of psychiatrists in our study was 63.24 ± 12.99, which appears to be much lower than those reported in previous studies (Rey et al., 2004; Heponiemi et al., 2014; Baumgardt et al., 2015). Further, regression analyses showed that higher job satisfaction was significantly associated with lower turnover intention. In addition, consistent with prior investigations (Linzer et al., 2000; Opoku and Apenteng, 2014; Lu et al., 2016; Meng et al., 2018; Zhang et al., 2018), more than half of psychiatrists were not satisfied with their current income, and we found that income satisfaction was significantly associated with lower turnover intention. The survey shows that the average monthly income of psychiatrists in 2020 was 12,144 RMBs (equivalent to 1908.65 USDs), which is much lower than that of psychiatrists in developed countries. This suggests that hospital managers can reduce the mobility of psychiatrists by improving doctors’ job satisfaction and salary.

### Association of Turnover Intention With Insomnia

Approximately 70% of psychiatrists reported insomnia, which was a surprisingly high rate compared to the prevalence rate of insomnia among the general population in China (15%; Cao et al., 2017). Moreover, the prevalence rate of insomnia among psychiatrists was significantly higher than that of medical staff during the COVID-19 pandemic (38.9%; Pappa et al., 2020). The higher rate of insomnia found in our study may be partially because we used less stringent criteria to assess insomnia (i.e., at least once over the past month). Nonetheless, insomnia can increase doctors’ anxiety, depression, traffic accidents, and impaired immune function (Taylor et al., 2003). Sufficient sleep is essential for mental and physical health, especially for medical staff who provide direct patient care. However, our survey found that the prevalence of insomnia among psychiatrists is significantly higher, which may be due to the particularity of the population they serve. For example, they often face patients with mental disorders, such as anxiety, insomnia, depression, and schizophrenia. In addition, there is a critical shortage of psychiatrists in China, so the increased demands placed on them in terms of night shifts and workload will undoubtedly result in insomnia being more prevalent among practicing psychiatrists. Importantly, we found that psychiatrists with insomnia were more likely to have turnover intention. This finding highlights the importance of improving sleep conditions of psychiatrists, which may help reduce the turnover intention rate of psychiatric medical staff.

## Limitations

This study has several limitations. First, we only investigated participants’ intention to leave their current employment, but we did not examine the nature of their intention—whether to work at another hospital as a psychiatrist, to work as a doctor in another department, or to leave psychiatry/medicine entirely. Additionally, as some studies suggest, turnover intention is often situational and reflective of employees’ frustration; as such, it is unclear what percentage of the participants who endorsed a turnover intention will leave their job. Second, the survey question assessing insomnia used a single-item standardized question, and we did not utilize more structured instruments, such as the Pittsburgh Sleep Quality Index (Buysse et al., 1989). Third, the psychiatrists included in this study were all from tertiary hospitals, and the results may not generalize to psychiatrists in other types of hospitals. Fourth, this study utilized a cross-sectional design, and thus we cannot draw causal conclusions between various factors and turnover intention. Finally, some questions in the survey may be vague and open to different interpretations, such as the experience of frontline work with COVID-19, which may affect our findings.

## Conclusion

This large-sample, national survey of psychiatrists working in tertiary hospitals showed that, during the COVID-19 pandemic, one-fifth of psychiatrists seriously considered leaving their employment in the next year. Primary factors that contributed to this intention to leave are insomnia, heavy workload, and involvement in frontline work against COVID-19. Policymakers and hospital administrators need to be aware of the potential challenges to workforce sustainability and address the concerns of Chinese psychiatrists through structural changes in the workplace. It is also necessary to provide effective psychological counseling to the frontline workers who participate in the fight against COVID-19 to empower them to cope effectively with the stressors they encounter to reduce people’s risk for workplace attrition and help ensure an optimal workforce.

## Data Availability Statement

The original contributions presented in the study are included in the article/supplementary material; further inquiries can be directed to the corresponding authors.

## Ethics Statement

The studies involving human participants were reviewed and approved by the Chaohu Hospital of Anhui Medical University. The patients/participants provided their written informed consent to participate in this study.

## Author Contributions

FJ, HL, and Y-lT: study design. YY, TL, YL, LX, LZ, and ML: collection, analyses, and interpretation of data. YY: drafting of the manuscript. NJK, DYL, and Y-lT: critical revision of the manuscript. All authors contributed to the article and approved the submitted version.

## Funding

The study was supported by the National Clinical Key Specialty Project Foundation (CN) and the Beijing Medical and Health Foundation (grant no. MH180924).

## Conflict of Interest

The authors declare that the research was conducted in the absence of any commercial or financial relationships that could be construed as a potential conflict of interest.

## Publisher’s Note

All claims expressed in this article are solely those of the authors and do not necessarily represent those of their affiliated organizations, or those of the publisher, the editors and the reviewers. Any product that may be evaluated in this article, or claim that may be made by its manufacturer, is not guaranteed or endorsed by the publisher.
